# Reviewer acknowledgement 2015

**DOI:** 10.1186/s13148-015-0061-9

**Published:** 2015-03-14

**Authors:** Ulrich Mahlknecht

**Affiliations:** Department for Internal Medicine - Hematology/Oncology, St. Lukas Klinik, Solingen, Germany; Department of Internal Medicine, Division of Immunotherapy and Gene Therapy, Saarland University Medical Center, Homburg/Saar, Germany

## Abstract

The Editors of *Clinical Epigenetics* would like to thank all our reviewers who have contributed to the journal in volume 6 (2014).

**Antonella Agodi**

Italy

**Nita Ahuja**

United States of America

**Hasan Aksit**

Turkey

**Anna Alisi**

Italy

**Lucia Altucci**

Italy

**Denise Andia**

Brazil

**Sergi Aranda**

Spain

**Amy Au**

Australia

**Shairaz Baksh**

Canada

**Anna-Maria Barciszewska**

Poland

**Rosaria Benedetti**

Italy

**Maria Berdasco**

Spain

**David Bernard**

France

**Bhavana Bhatnagar**

United States of America

**Karol Bomsztyk**

United States of America

**Carrie Breton**

United States of America

**Robert Brown**

United Kingdom

**Bethany Buck-Koehntop**

United States of America

**Hyang-Min Byun**

United States of America

**Bernard Callus**

Australia

**Gabriel Capella**

Spain

**Martin Carroll**

United States of America

**Paolo Casali**

United States of America

**Po-Yuan Chang**

Taiwan

**Isabelle Chemin**

France

**Jing Chen**

United States of America

**Wantao Chen**

China

**Yangchao Chen**

Hong Kong

**Yuen Yee Cheng**

Australia

**Nan-Haw Chow**

Taiwan

**Brock Christensen**

United States of America

**Jeffrey Craig**

Australia

**Magali Cucchiarini**

Germany

**Hengmi Cui**

United States of America

**Roderick Dashwood**

United States of America

**Jessica de Greef**

United States of America

**George Dedoussis**

Greece

**Floriana Della Ragione**

Italy

**Carmela Dell'aversana**

Italy

**Emma Dempster**

United Kingdom

**Paula Desplats**

United States of America

**Stella Dracheva**

United States of America

**Julie Dumonceaux**

France

**Thomas Eggermann**

Germany

**Mattias Ekstedt**

Sweden

**Tomas Ekström**

Sweden

**Osman El-Maarri**

Germany

**Jesus Espada**

Spain

**Mario Fdez. Fraga**

Spain

**Alexey Fedulov**

United States of America

**Rebecca Fry**

United States of America

**Hiroshi Furukawa**

Japan

**Philippe Georgel**

United States of America

**Santosh Ghosh**

United States of America

**Marcus Gomes**

Brazil

**Zhiyun Gong**

China

**Steven Gray**

Ireland

**David Guerrero**

Spain

**Sonia Guil**

Spain

**Sanjay Gupta**

United States of America

**Qin Han**

China

**Alexander Haslberger**

Austria

**Martin Hasselblatt**

Germany

**Danielle Heideman**

Netherlands

**Luke Hesson**

Australia

**Kinjal Hew**

United States of America

**Yu-Mei Hsueh**

Taiwan

**Hailiang Huang**

United States of America

**Yun Huang**

United States of America

**Mazhar Iqbal**

Pakistan

**Jean-Pierre Issa**

United States of America

**Nikolai Ivanov**

Switzerland

**Vincent Jaddoe**

Netherlands

**Jaroslav Jelinek**

United States of America

**Carmen Jeronimo**

Portugal

**Yideng Jiang**

China

**Guanghui Jin**

China

**Ricky Johnstone**

Australia

**Suh-Hang Juo**

Taiwan

**Claudia Kappen**

United States of America

**Atsushi Kasai**

Japan

**Wahab Khan**

Canada

**Seong Who Kim**

South Korea

**Mark Kirschbaum**

United States of America

**Gaspar Kitange**

United States of America

**Yutaka Kondo**

Japan

**Igor Koturbash**

United States of America

**Jose Miguel Laffita Mesa**

Cuba

**Janine LaSalle**

United States of America

**Elena Lesma**

Italy

**Eric Letouzé**

France

**Bin Li**

China

**Hsin-Pai Li**

Taiwan

**Jonathan Licht**

United States of America

**Charlotte Ling**

Sweden

**Yi-Wen Liu**

Taiwan

**Qianjin Lu**

China

**Deborah Mackay**

United Kingdom

**Luca Magnani**

United Kingdom

**Philip Marsden**

Canada

**Deborah Marsh**

Australia

**Carmen Marsit**

United States of America

**Daniel Martinez-Arguelles**

Canada

**Maria Matarazzo**

Italy

**Hisahiro Matsubara**

Japan

**Richard Meehan**

United Kingdom

**Alexander Michels**

United States of America

**Montserrat Mila**

Spain

**Daniel Miller**

United States of America

**Monica Miozzo**

Italy

**Pradyumna Mishra**

India

**Richard Momparler**

Canada

**Eva Morales**

Spain

**Mo Motamedi**

United States of America

**Pamela Munster**

United States of America

**Martinet Nadine**

France

**Kazuhiko Nakabayashi**

Japan

**Kazuaki Nakamura**

Japan

**Rajeswari Narayanappa**

India

**Ruta Navakauskiene**

Lithuania

**Clara Nervi**

Italy

**Torbjörn Nilsson**

Sweden

**Houtan Noushmehr**

Brazil

**Margarete Odenthal**

Germany

**Shuji Ogino**

United States of America

**Sam Oh**

United States of America

**Rieko Ohki**

Japan

**Joyce Ohm**

United States of America

**Jaroslaw Paluszczak**

Poland

**Vassilios Papadopoulos**

Canada

**Arpad Patai**

Hungary

**Mattia Pelizzola**

Italy

**Palsamy Periyasamy**

United States of America

**Marcello Persico**

Italy

**W. Jeffrey Petty**

United States of America

**Carmen Pheiffer**

South Africa

**Ruth Pidsley**

United Kingdom

**Christoph Plass**

Germany

**Torsten Plosch**

Netherlands

**Jacek Polosak**

Poland

**Deepa Rastogi**

United States of America

**Noel Raynal**

Canada

**Stella Marie Reamon-Buettner**

Germany

**Andrea Riccio**

Italy

**Tina Rönn**

Sweden

**Rossella Rota**

Italy

**Marianne Rots**

Netherlands

**Lisa Russell**

United Kingdom

**Richard Saffery**

Australia

**Yan Sanders**

United States of America

**Carmen Sapienza**

United States of America

**Primo Schär**

Switzerland

**Carlo Selmi**

United States of America

**Alexander Semmler**

Switzerland

**Huidong Shi**

United States of America

**David Simar**

Australia

**Roderick Slieker**

Netherlands

**Alicia Smith**

United States of America

**Tamar Sofer**

United States of America

**Tijana Stankovic**

Serbia

**Renske Steenbergen**

Netherlands

**Michael Steiner**

New Zealand

**David Stewart**

Canada

**Cong Sun**

Australia

**Yuan Sun**

United States of America

**Hua Tang**

China

**Kai-Fu Tang**

China

**Wan Yee Tang**

United States of America

**Andrew Teschendorff**

China

**Mathewos Tessema**

United States of America

**Kulbhushan Tikoo**

India

**Yasushi Toh**

Japan

**Trygve Tollefsbol**

United States of America

**Stefania Tommasi**

Italy

**Jérôme Torrisani**

France

**Rossella Tupler**

United States of America

**Toshikazu Ushijima**

Japan

**Fatima Valdes Mora**

Australia

**Nicholas Ventham**

United Kingdom

**Mukesh Verma**

India

**Paolo Vineis**

United Kingdom

**Sarah Voisin**

Sweden

**Wolfgang Wagner**

Germany

**Thomas Wilhelm**

United Kingdom

**Maria Wing**

United States of America

**Bea Wisman**

Netherlands

**Olaf Witt**

Germany

**Yu Mi Woo**

South Korea

**Tongjing Xing**

China

**Jin-Chen Yang**

United States of America

**Qiwei Yang**

United States of America

**Nadia Zaffaroni**

Italy

**Kai Zhang**

China

**Yan Zheng**

United States of America

**Vlatka Zoldos**

Croatia

